# Effect of artificial tears on dynamic optical quality in patients with dry eye disease

**DOI:** 10.1186/s12886-022-02280-7

**Published:** 2022-02-10

**Authors:** Zhenyu Wei, Yuandong Su, Guanyu Su, Christophe Baudouin, Antoine Labbé, Qingfeng Liang

**Affiliations:** 1 Beijing Institute of Ophthalmology, Beijing Tongren Eye Center, Beijing Tongren Hospital, Capital Medical University, Beijing Key Laboratory of Ophthalmology and Visual Sciences, Beijing, 100005 China; 2 Quinze-Vingts National Ophthalmology Hospital, IHU FOReSIGHT Paris and Versailles Saint-Quentin-en-Yvelines University, Versailles, France; 3 INSERM, U968, F-75012 Paris, France; 4 UPMC Univ Paris 06, UMR_S 968, Institut de la Vision, F-75012 Paris, France; 5 CNRS, UMR_7210, F-75012 Paris, France

**Keywords:** Objective scatter index, Tear film, Artificial tear, Dry eye

## Abstract

**Background:**

In clinical practice, fluctuating vision or decreased quality of vision is a common complaint in DED patients. Our study was designed to investigate the change in dynamic optical quality in dry eye patients after the use of artificial tears.

**Methods:**

Fifty-nine patients with dry eye disease (DED) and 31 control subjects were included in this prospective case-control study. There was no significant difference in age and sex between these two groups (*P* = 0.342, *P* = 0.847, respectively). Clinical evaluation of the ocular surface included Ocular Surface Disease Index (OSDI), tear film break-up time (TBUT), lipid layer thickness (LLT), and Schirmer I test. DED patients were divided into two groups, mild (31 patients) and severe (28 patients). The optical quality of the tear film was measured with the Optical Quality Analysis System (OQAS) using the mean objective scatter index (mean OSI), standard deviation of objective scatter index (SD-OSI) and modulation transfer function cut-off (MTF cut-off). After baseline examinations, one drop of artificial tears (ATs, carboxymethylcellulose ophthalmic solution, 0.5%) was instilled in both eyes, and optical quality parameters were measured again at 5 and 30 min following application of ATs.

**Results:**

At baseline, the mean OSI was higher in the DED group (0.95 ± 0.54) than in controls (0.54 ± 0.23, *P* < 0.001). The SD-OSI was also significantly increased in DED patients (0.44 ± 0.71) compared to control subjects (0.12 ± 0.06, *P* = 0.003). Five minutes after AT instillation, mean OSI and SD-OSI decreased significantly in severe DED patients *(P* = 0.044; *P* = 0.018), remained unchanged in mild DED patients, and increased in the control group (*P* = 0.019; *P* < 0.001). Thirty minutes after AT instillation, no significant difference in optical quality parameters was observed among the three groups.

**Conclusion:**

The effect of ATs on optical quality in patients with DED may differ according to the severity of the disease. Measurement of optical quality might be a promising tool to evaluate the effects of various ATs and possibly individualize treatment in DED patients.

## Background

Dry eye disease (DED) is a multifactorial disease of the ocular surface, characterized by an unstable tear film associated with symptoms of ocular surface irritation and visual impairment [1]. In addition to its nutritive and mechanical roles on the ocular surface, the tear film can mask irregularities of the corneal epithelium, and, consequently, it is a central component of the refractive system of the eye. Break-up of the precorneal tear film can cause irregular astigmatism in excess of 1 diopter [2]. In clinical practice, fluctuating vision or decreased quality of vision are common complaints in DED patients. Several studies [3–8] have shown that DED patients may experience transient increases in higher-order aberrations (HOA) and objective scatter index (OSI). A previous study by our group also demonstrated that OSI fluctuations were significantly increased in eyes with DED, and the degree of fluctuations was correlated with the severity of the DED [4]. Habay et al. showed similar results, with both the mean value and standard deviation of the OSI significantly decreased in severe dry eye patients [9].

Artificial tears (ATs), as standard first-line therapy, are typically used for DED patients, especially for aqueous deficient dry eye. Water-retentive polymers such as carboxymethylcellulose (CMC) have long been incorporated in AT formulations to supplement the aqueous, maintain and/or restore the stability of the tear film, and protect the ocular surface [10]. Although ATs decrease ocular surface symptoms in DED patients, some patients complain of transient blurry vision or decreased visual acuity after AT use. Consequently, several studies evaluating optical quality after instilling artificial tear drops have been performed, but the results have been controversial. Some studies [11] have reported that visual quality, as reflected by higher order aberrations (HOA), deteriorates after instilling artificial tear drops, while some studies have revealed that visual quality improves and is sustained for some time after instillation [12]. Several reasons might explain these conflicting results, including non-objective methods of evaluation or differing severities of DED. Consequently, in the present study, we used the double pass Optical Quality Analysis System (OQAS) to evaluate the visual quality of DED patients with differing severities and normal controls after the use of 0.5% CMC ATs. The goal was to evaluate the effect of ATs on optical quality in DED patients.

## Methods

### Subjects

This study was conducted at the Beijing Tongren Hospital with the approval of the hospital’s Medical Ethics Committee (TREC-2017-KY021). All subjects were informed of the goals of the study, and their consent was obtained in accordance with the declaration of Helsinki. A total of 59 DED patients (36 women and 23 men; mean age 36.05 ± 12.56 years; range 18–72 years) were consecutively recruited from July 2018 to August 2019. In keeping with the consensus report by the International Dry Eye Workshop (2007) [13], the inclusion criteria for the DED group were as follows: (1) age > 18 years; (2) Ocular Surface Disease Index (OSDI) [14] score greater than 12; (3) tear film break-up time (TBUT) less than 10 s; (4) Schirmer I test at 5 min less than 10 mm. According to the severity-grading scheme of the 2007 Workshop [13], DED patients were divided into two groups (mild and severe). The severe DED group (28 patients) included patients with Schirmer I test ≤5 mm. The mild DED group (31 patients) consisted of patients with Schirmer I test between 5 and 10 mm. Thirty-one control subjects (21 women and 10 men; mean age 38.13 ± 13.79 years, range 18–67 years) were also recruited, with Schirmer I test results exceeding 10 mm at 5 min, TBUT above 10 s and an OSDI score below 13. The exclusion criteria for both groups were as follows: (1) age < 18 years; (2) subjects unable to complete the questionnaire or understand the procedures; (3) best corrected visual acuity less than 20/20; (4) presence of ocular or systemic disease or the use of topical or systemic medications that may affect the ocular surface, and (5) previous history of eye surgery or contact lens wear.

### Clinical evaluation

Each subject underwent a quantification of ocular surface symptoms with the Ocular Surface Disease Index (OSDI) questionnaire (range 0–100) [14]. Optical quality and ocular surface examinations were then performed in the following order: double-pass optical quality analysis, lipid layer thickness measurement (LLT), TBUT and Schirmer I test.

The lipid layer thickness was measured with the Lipiview II® device (Tear science, Morrisville, NC, USA). The interferometric color unit (ICU) value reflected the local LLT with 1 ICU equivalent to 1 nm of lipid layer thickness. TBUT was measured using sterile fluorescein strips impregnated with 0.6 mg fluorescein sodium (Alcon Laboratories, St. Louis, MO, USA). After applying 50 μL of normal saline solution to the paper strip, it was touched to the inferior fornix. The interval between a complete blink and the appearance of the first dry spot was noted. TBUT was measured 3 times, and the mean calculated. The Schirmer’s test was performed without anesthesia after having the patient’s eyes closed for 5 min.

### Objective scatter index measurement

**Fig. 1:**
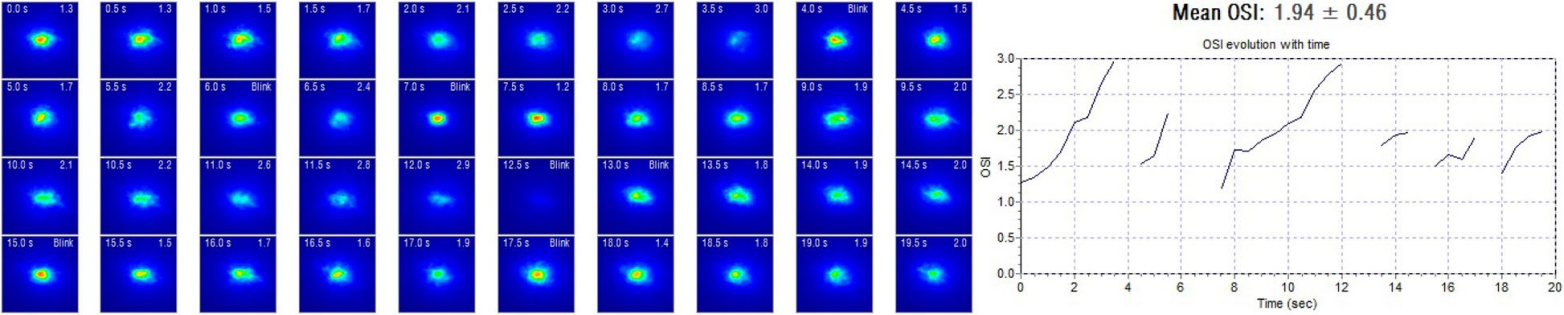
The OQAS device records a 20-s examination with OSI measurement every 0.5 s. Image mode (left) presented the change in OSI over time. Line graph (right) provided the definite OSI evolution with time, whose data were analyzed from the left

The Optical Quality Analysis System (OQAS-II, Visiometrics, Tarrasa, Spain), was used to evaluate aberrations and intraocular scatter with the 2 following parameters: objective scatter index (OSI) and modulation transfer function cut-off (MTF cut-off). The OSI is defined as the ratio between the integrated light in the peripheral ring and the central peak of the double-pass (DP) image. It represents the impact of aberration and scattering on the DP image. The MTF cut-off is the cut-off point on the x-axis of the MTF curve, which can be directly calculated from the point spread function. The MTF cut-off reflects the highest spatial frequencies that can be distinguished by patients’ eyes at which the MTF reaches the lowest contrast of 1%. The subjects were tested with their best correction and instructed to blink normally during the double-pass analysis. The “Tear Film Analysis” program of the commercially available OQAS device was used to record dynamic changes in OSI values. This program consisted of a 20-s examination with OSI measurement every 0.5 s, providing a graph showing the change in OSI over time (Fig. 1). The pupil size was set at 7 mm to cover the majority of the tear film. After measurement, the mean value and standard deviation of the 20-s OSI was recorded as “mean OSI” and “SD OSI,” respectively.

After baseline examination, the effect of artificial tears was studied on the following day. One drop of artificial tears (carboxymethylcellulose ophthalmic solution 0.5%, Refresh Plus, Allergan Pharmaceuticals, Ireland) was instilled in both eyes. Five and thirty minutes after application, the OQAS examinations were performed again in all subjects. The difference between the mean OSI at baseline and at 5 and 30 min (∆ OSI_5min_, ∆ OSI_30min_) were analyzed, and the relationship between ∆ OSI and DED severity was evaluated.

### Statistical analysis

Statistical analysis was performed with SPSS for Windows, version 22.0 (SPSS Inc., Chicago, IL, USA). For each subject, both eyes were tested, and the right eye was selected for analysis. All values were expressed as mean ± SD. The normality of the data was analyzed using the Shapiro-Wilk test. The normal data were compared before and after treatment with the paired *t*-test, and the non-normal data were used for the paired rank-sum test. A Bonferroni correction of multiple comparisons was included. The generalized estimation equation was used to compare the repeated measurement data at each time point and within each treatment group. The Spearman correlation test was used to evaluate the correlation between the visual quality parameters and dry eye clinical tests. A *P* < 0.05 was considered statistically significant.

## Results

### Subject clinical characteristics

There was no difference with respect to sex (*P* = 0.342) or age (*P* = 0.847) between the DED (mild and severe group) and control groups. Regarding ocular surface symptoms, DED patients had more symptoms (OSDI score: mild group 24.04 ± 5.22, severe group 52.66 ± 14.10) than normal controls (8.67 ± 3.29) (all *P* < 0.001). In addition, the TBUT in the mild (4.05 ± 1.08 s) and severe (3.17 ± 0.99 s) DED groups was significantly less than in normal controls (11.84 ± 3.12 s), (*P* < 0.001). Compared to control subjects (16.26 ± 4.98 mm), the Schirmer I test was lower in the mild (8.48 ± 2.74 mm, *P* < 0.001) and severe group (3.83 ± 1.25 mm; *P* < 0.001). The mild and severe DED patients had significantly lower LLT (59.29 ± 15.53 nm, *P* = 0.015; 54.14 ± 16.84 nm, *P* = 0.001) compared to normal controls (70.81 ± 19.33 nm). However, no statistical difference in LLT was observed between the two DED groups (*P* = 0.223) (Table 1).

**Table 1:** Demographic information and baseline results of ocular surface tests

	Control	Mild ADDE	Severe ADDE	***P*** value	***P*** value (subgroup comparison)		
	(31 eyes)	(28 eyes)	(31 eyes)	within groups	control vs mild	control vs severe	mild vs severe
**Male**	10	13	10	0.342	0.269	0.785	0.171
**Female**	21	15	21				
**Age, y**	38.13±13.79	37.71±13.43	36.35±14.40	0.847	0.982	0.678	0.564
**OSDI, units**	8.67±3.29	24.04±5.22	52.66±14.10	<0.001	<0.001	<0.001	<0.001
**TBUT, s**	11.84±3.12	4.05±1.09	3.17±0.99	0.002	<0.001	<0.001	0.024
**Schirmer I, mm**	16.26±4.98	8.48±2.74	3.83±1.25	<0.001	<0.001	<0.001	<0.001
**LLT, nm**	70.81±19.33	59.29±15.33	54.14±16.84	0.015	0.009	<0.001	0.452

*Note*: Data were expressed as the means ± SD. Due to multiple comparisons, the inspection level was corrected to 0.0125

### Optical quality analysis at baseline

**Fig. 2:**
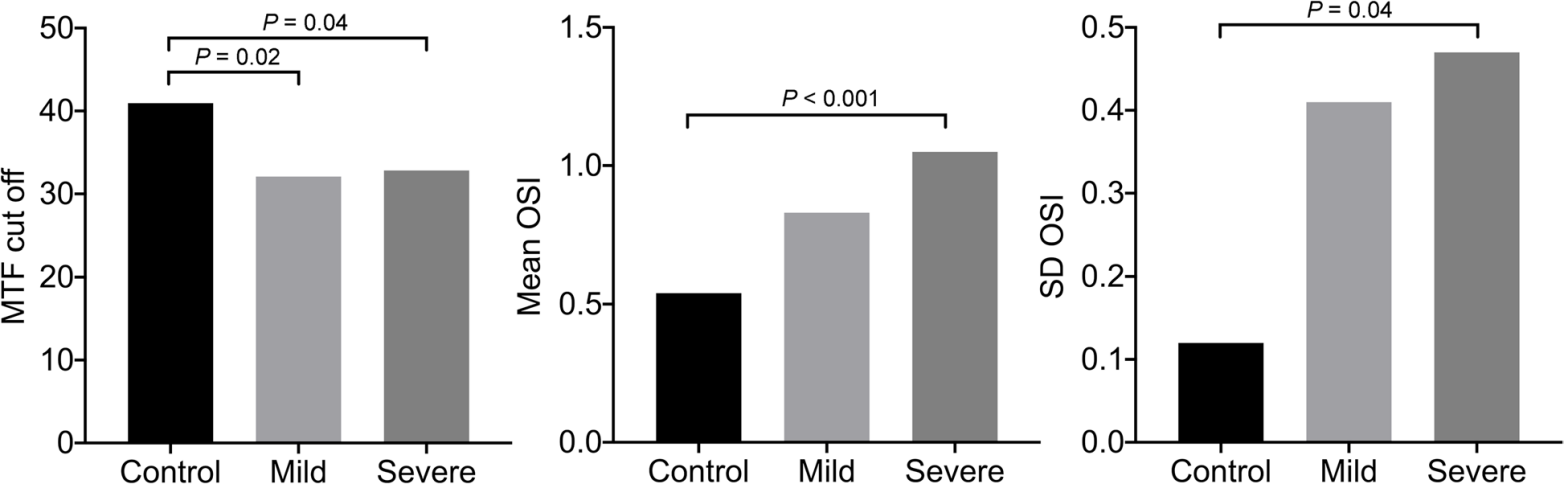
Baseline analysis of optical quality in DED patients and control subjects

The results of the optical quality examinations at baseline showed that the MTF cut-off value in the mild (32.11 ± 11.16, *P* = 0.003) and severe (32.84 ± 11.84, *P* = 0.013) groups was significantly decreased compared to normal subjects (40.96 ± 9.07). The mean OSI in the mild group (0.83 ± 0.38, *P* = 0.001) and severe group (1.05 ± 0.65, *P* < 0.001) were significantly increased compared to normal subjects (0.54 ± 0.23). There was no statistical difference between the mild and severe DED groups for the mean OSI (*P* = 0.114). The SD OSI value in the mild (0.41 ± 0.69, *P* = 0.048) and severe DED group (0.47 ± 0.72, *P* = 0.011) was significantly higher than in normal subjects (0.12 ± 0.06), and there was also no significant difference between the mild and severe groups (*P* = 0.671) (Fig. 2).

### Changes in optical quality after AT application

**Fig. 3:**
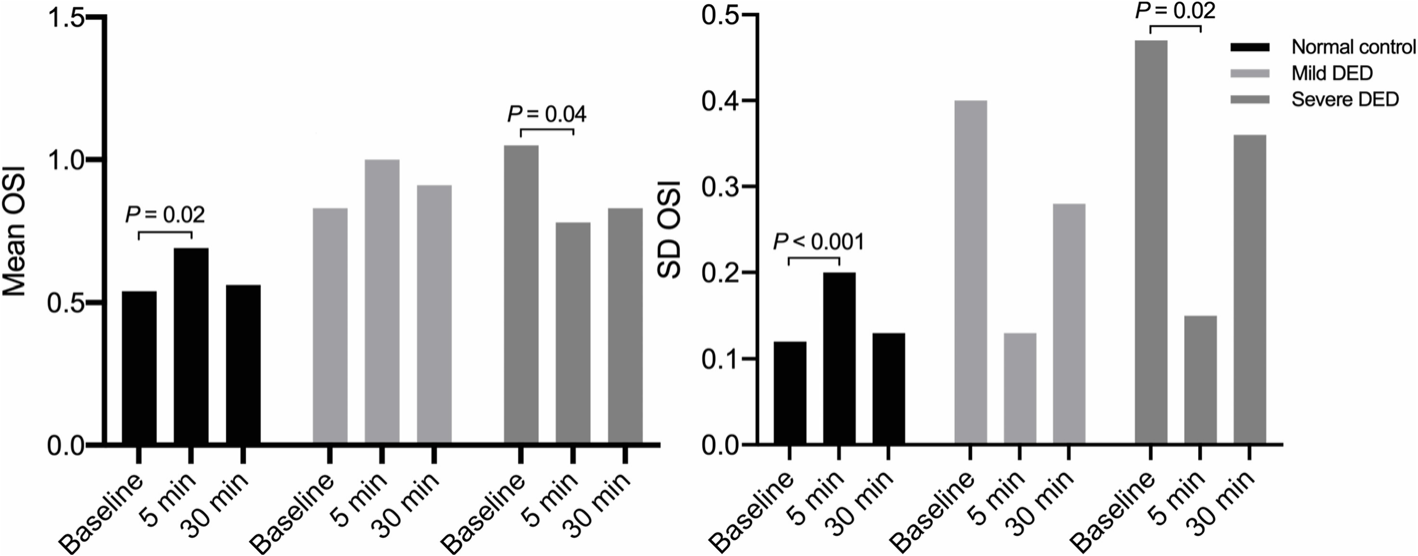
Change in mean OSI and SD OSI from baseline to 5 min and 30 min after AT instillation

Five minutes after AT instillation, the mean and SD of the OSI increased significantly in the control group, from 0.54 ± 0.23 and 0.12 ± 0.06 at baseline to 0.69 ± 0.27 and 0.20 ± 0.10, respectively (*P* = 0.019 and *P* < 0.001). In the mild group, the mean OSI (1.00 ± 0.48) and the SD OSI (0.13 ± 0.05) did not change significantly compared to the baseline results (0.83 ± 0.38, *P* = 0.155; 0.40 ± 0.70, *P* = 0.066). In the severe group, both the mean OSI (0.78 ± 0.36 vs. 1.05 ± 0.65, *P* = 0.044) and the SD OSI (0.15 ± 0.08 vs. 0.47 ± 0.72, *P* = 0.018) decreased significantly at 5 min after AT application. Inversely, compared to baseline values, the parameters of mean and SD OSI had no significant difference at 30 min after AT instillation in any of the groups (Fig. 3).

### Correlation between change in optical quality and clinical tests

Within the DED groups, there were significant negative correlations between TBUT and mean OSI (*r* = − 0.329, *P* = 0.011), and TBUT and SD-OSI (r = − 0.330, *P* = 0.011). TBUT was also positively correlated with the ∆OSI_5min_ (*r* = 0.261, *P* = 0.045). Similarly to the TBUT, the Schirmer I test was negatively correlated with the mean OSI (*r* = − 0.251, *P* = 0.050) and SD-OSI (r = − 0.349, *P* = 0.007) and positively correlated with the ∆OSI_5min_ (*r* = 0.457, *P* = 0.000) (Table 2).

**Table 2:** Optical quality comparison of participants before after artificial tears application

	n	Mean OSI			SD OSI		
		Baseline	5 min	30 min	Baseline	5 min	30 min
**Normal control**	31	0.54±0.23	0.69±0.27	0.56±0.29	0.12±0.06	0.20±0.10	0.13±0.05
t value			-2.418	-0.338		-3.802	-0.481
*P* value			0.019	0.737		0.000	0.632
**Mild ADDE**	28	0.83±0.38	1.00±0.48	0.91±0.53	0.40±0.70	0.13±0.05	0.28±0.27
t value			-1.444	-0.597		1.966	0.848
*P* value			0.155	0.553		0.066	0.402
**Severe ADDE**	31	1.05±0.65	0.78±0.36	0.83±0.76	0.47±0.72	0.15±0.08	0.36±0.38
t value			2.073	1.214		2.508	0.752
*P* value			0.044	0.299		0.018	0.455

*Note*: Data were expressed as the means ± SD. "Mean OSI" and "SD OSI" refer to the mean value and standard deviation of the 20-second OSI

## Discussion

Blurred vision and visual fluctuations are significant complaints in DED patients. Previous studies have found that the visual disturbances in DED are related to the unstable tear film [3–5]. As a component of the refractive system of the eye, the tear film covers the ocular surface and corrects for irregularities in the corneal epithelium [2]. The optical quality of the tear film has been considered as a parameter for evaluation of the stability of the tear film and the severity of DED [15–17]. Similarly, to previous studies, our study showed that optical quality parameters (MTF cut-off, mean OSI and SD-OSI) were changed in DED patients and these changes are correlated to DED severity.

When considering the effects of ATs on DED patients, the results of previous studies evaluating quality of vision have been controversial [18–24]. In 1993, Rieger et al. [22] suggested that ATs could improve contrast sensitivity for severe DED patients. Misaki et al. [23] detected changes in visual acuity with different concentrations of ATs and found no significant difference in mild patients’ visual acuity 5 min after AT application. However, these two studies only focused on the changes in visual quality a few minutes after AT instillation. Tung et al. [24] presented differing results, with a decrease in visual quality 5 min after AT instillation, but they did not analyze different DED severity groups. In the present study, 5 min after instillation, the severe and mild dry eye and normal subjects showed differing results. An increase in optical quality was observed in the severe group, there was no change in the mild group, and significant degradation of optical quality was shown in normal subjects. The improvement in optical quality may be related to the severity of tear volume deficiency. In the severe group, in whom the Schirmer I test was less than 5 mm, artificial tears can quickly restore the aqueous layer and the optical role of the tear film on the ocular surface. In the mild group, a trend toward improvement in the SD OSI was observed, but these changes were not significant.

In our study, the OSI mean and SD showed no significant difference at 30 min after AT instillation in any of the groups. These findings are consistent with previous studies showing a rapid return of tear film biomarkers to baseline after AT instillation. In a study evaluating the tear meniscus depth and height by optical coherence tomography (OCT), Carracedo et al. [25] found no statistically significant difference in tear volume and stability between baseline and 20 min after AT instillation. The same results were observed by Napoli et al. [26] In their study, 20 min after instillation of ATs, the tear film thickness and tear meniscus volume returned to baseline values in all groups. Moreover, in the study by Koh, there was a correlation between wavefront error and tear meniscus height [27].

In our study, the OSI mean and SD values increased after AT application in the control group, which is consistent with previous studies [24, 28]. Our first hypothesis is that the volume of ATs might dilute the original tear film in normal patients and change the biochemical properties such as viscosity or refractive index. This may lead to tear film instability and subsequently to an increased tear meniscus, as revealed by Shizuka et al. [28] In DED patients, the key optical problem is tear deficiency on the ocular surface. Consequently, it might be replenished by ATs, with resultant improvement in visual quality. Nevertheless, in the present study, after AT instillation, the improved mean OSI in severe DED patients was more obvious than in normal subjects at baseline. Consequently, the hypothesis of the lubricant-only role of ATs might not completely explain this result. Another hypothesis could be the role of osmolarity. The cell membrane of corneal epithelial cells is a semi-permeable membrane allowing fluid transport within the intercellular space depending on particle concentration. With increased evaporation, the tear volume decreases, and the osmolarity of the tear film increases. Previous studies have shown that tear osmolarity in mild and severe DED patients is significantly increased (approximately 315 mOsm/L and 336 mOsm/L, respectively) compared to normal controls (302 mOsm/L) [29]. Most types of ATs for DED treatment have been hypo-osmolar [30]. Similarly, the AT used in the present study (Refresh Plus) has an osmolarity of 294 ± 2 mOsm/L, lower than that of the tear film [30]. Therefore, 5 min after instillation, the ocular surface osmolarity may be equilibrated to a new lower level. In DED patients, the tear volume was replenished and tear film osmolarity decreased to the normal level, leading to an increase in tear film stability and visual quality. The effect of ATs on normal subjects might be different. The osmolarity of the tear film might decrease due to dilution by hypotonic ATs. Following the osmotic pressure gradient, the corneal epithelial cells might become edematous, characterized by swelling of the epithelial cells and distension of the intercellular spaces [31], causing light to be scattered and visual quality to be decreased.

Although there are several limitations to the present study, including using Schirmer test as a grading parameter in our study. Other parameters (i.e. ocular surface staining score, OSDI) may also work for the dynamic optical quality. Another limitation was the use of only one type of ATs and the lack of characterization of the type of DED (i.e. meibomian gland dysfunction vs. aqueous deficiency), we showed that the effect of ocular lubricants on visual quality may vary according to the severity of DED. There also had a limitation that the interference of the examinations, especially Schirmer’s testing, with the tear film. To minimize the impact of prior testing, we added a control group and examined the effect of artificial tears on the following day.

The objective evaluation of optical quality with the OQAS seems to be an interesting clinical parameter that can be easily associated with and compared to the classic clinical signs used in DED. It may be a promising tool in evaluating the effects of various ATs and possibly individualizing treatment in DED patients, avoiding those preparations which cause the most blurred vision and favoring those which improve quality of vision over a longer period.

## Conclusions

In summary, our data indicate that optical quality may be improved significantly 5 min after AT instillation in severe DED cases, unchanged in mild DED cases, and decreased in the normal controls. For this reason, measurement of optical quality could be a promising tool to evaluate the effects of ATs in DED patients. The data support our hypothesis that the visual quality of DED patients may be influenced by AT application, and these differences may depend on the severity of DED. To make up for the lack of current research, our future studies might focus on the effect of various ATs and dynamic changes in optical quality. We might also concentrate on patients with evaporative dry eye.

Our present and future studies might eventually help DED patients individualize therapy in terms of selecting the most beneficial artificial tear. This study may also be significant to the improvement of the components and technology of artificial tears.

## Data Availability

The datasets used and analyzed in this study are available from the corresponding author on reasonable request.

## Abbreviations

ATs: Artificial tears
DED: Dry eye disease
DP: Double-pass
HOA: Higher-order aberrations
ICU: Interferometric color unit
LLT: Lipid layer thickness
Mean OSI: Mean Objective Scatter Index
MTF cut-off: Modulation transfer function cut-off
OSDI: Ocular Surface Disease Index
OSI: Objective scatter index
OQAS: Optical Quality Analysis System
SD-OSI: Standard deviation of objective scatter index
TBUT: Tear film break-up time
OCT: optical coherence tomography
